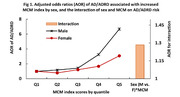# Sex Differences in Comorbidity Patterns and the Associations with Risk of Alzheimer’s Disease and AD‐Related Dementias among Adults Aged 65 and Older: Findings from the United States Medicare Current Beneficiaries Surveys, 2018‐2020

**DOI:** 10.1002/alz.089553

**Published:** 2025-01-09

**Authors:** Longjian Liu, Edward J Gracely, Nathalie S May, Rose Ann Rose Ann DiMaria‐Ghalili, Howard J Eisen

**Affiliations:** ^1^ Drexel University Dornsife School of Public Health, Philadelphia, PA USA; ^2^ Drexel University College of Medicine and Dornsife School of Public Health, Philadelphia, PA USA; ^3^ Drexel University College of Medicine, Philadelphia, PA USA; ^4^ Drexel University College of Nursing and Health Professions, Philadelphia, PA USA; ^5^ Thomas Jefferson University Hospital, Philadelphia, PA USA

## Abstract

**Background:**

To provide a nationally representative estimate of the burden of multiple comorbidities (MCM) among community‐dwelling Medicare beneficiaries with Alzheimer’s disease (AD) and AD related dementias (ADRD).

**Method:**

We analyzed national sampling data in Medicare beneficiaries aged ≥65 (n = 34,318) from the 2018‐2020 Medicare Current Beneficiaries Surveys. AD/ADRD and twelve chronic comorbidities (hypercholesterolemia, hypertension, diabetes, coronary heart disease, heart failure, stroke, depression, mental disorder, chronic kidney disease, chronic obstructive pulmonary disease, arthritis, and osteoporosis) were examined. A novel age‐adjusted regression weighted MCM index was developed by taking into account the relative impact of each comorbidity on the risk of AD/ADRD. Logistic regression models estimated adjusted odds ratios (AORs) of commodity and the MCM index for AD/ADRD, adjusting for sociodemographic, body mass index and behavior factors.

**Result:**

A total of 1,802 AD/ADRD cases (5.3%) were identified, representing an estimated 5,580,421 AD/ADRD cases in the nation’s Medicare beneficiaries. In the 65‐74 age group, the prevalence of AD/ADRD was 1.8% in males and 1.4% in female (p = 0.11), while in those aged ≥75, it was 6.1% in males and 7.6% in females (p = 0.001). AD/ADRD individuals more often had ≥4 comorbidities than those without AD/ADRD (52.6% vs. 30.9% in males, and 58.3% vs. 34.8% in females). In males, of the 12 comorbidities, depression exhibited the highest AOR (95%CI) at 4.62 (3.70‐5.78) for AD/ADRD risk, followed by mental disorder at 4.36 (2.88‐6.59), and stroke at 3.19 (2.51‐4.06). The same disorders were the top three in females, but in a different order, namely mental disorders at 4.79 (3.44‐6.68), stroke at 3.50 (2.79‐4.39), and depression at 2.92 (2.41‐3.54). An increase in MCM index score was associated with 2.13 (1.92‐2.37) higher AD/ADRD risk in males, and 1.65 (1.52‐1.80) higher risk in females.

**Conclusion:**

The study highlights that AD/ADRD poses a substantial public health burden in the nation, with older females experiencing a significantly higher prevalence of AD/ADRD. The association of sex and specific comorbidity profiles on AD/ADRD risk is evident, emphasizing the need for further studies of the disease mechanisms and the potentially targeted prevention strategies by addressing these factors.